# Utility of Centrifugation-Controlled Convective (C3) Flow for Rapid On-chip ELISA

**DOI:** 10.1038/s41598-019-56772-6

**Published:** 2019-12-27

**Authors:** Wilfred Espulgar, Tatsuro Tadokoro, Eiichi Tamiya, Masato Saito

**Affiliations:** 1 Department of Applied Physics, Osaka University, 2-1 Yamadaoka, Suita, 565-0871 Osaka Japan; 2 AIST, PhotoBIO-OIL, Photonics Center Osaka University P3 Bldg, 2-1 Yamadaoka, Suita, 565-0871 Osaka Japan

**Keywords:** Chemical engineering, Chemical engineering

## Abstract

Miniaturizing the enzyme-linked immunosorbent assay (ELISA) protocols in microfluidics is sought after by researchers for a rapid, high throughput screening, on-site diagnosis, and ease in operation for detection and quantification of biomarkers. Herein, we report the use of the centrifugation-controlled convective (C3) flow as an alternative method in fluid flow control in a ring-structured channel for enhanced on-chip ELISA. A system that consists of a rotating heater stage and a microfluidic disk chip has been developed and demonstrated to detect IgA. The ring-structured channel was partially filled with microbeads (250 µm in diameter) carrying the capture antibodies and the analyte solution was driven by thermal convection flow (50 µL/min) to promote the reaction. The remaining part of the circular channel without microbeads served as the observation area to measure the absorbance value of the labeled protein. Currently, the system is capable of conducting four reactions in parallel and can be performed within 30 min at 300 G. A detection limit of 6.16 ng/mL using 24 µL of target sample (IgA) was observed. By simply changing the capture antibodies, the system is expected to be versatile for other immunoassays.

## Introduction

Enzyme-linked immunosorbent assay (ELISA) is broadly used as a diagnostic tool in clinical applications for its precision, sensitivity, versatility, and quantifiability^1,2^. The quantification and monitoring of small molecules such as drug and metabolites, large proteins, nucleic acids, and even whole pathogens were made possible due to the sensitivity of the antibody-antigen interaction^3^. This provides a broad application in medical diagnosis, environmental measurement, and food safety evaluation. However, conventional ELISA protocols require multiple steps of adsorption, washing, and incubation of reagents. It’s labor-intensive and time-consuming which often takes several hours, that can even last up to 2 days to perform a single assay^4^. In addition, immuonagents used in ELISA are relatively expensive and usually done in a centralized lab with standard laboratory equipment. For health care administration, early and accurate detection is critical to increase survivability, improve disease management and treatment outcomes, and formulate preventive and personalized medicine^5–7^. Thus, a strong demand remains for immunoassays with shorter turnaround time and low cost with acceptable or, if not, improved sensitivities and accuracy.

The long incubation time required in typical immunoassay is attributed to the inefficient mass transport. Although the immunoreaction is relatively rapid, the immunoagents need to migrate from a solution to the surface where the conjugation occurs^8^. Microfluidics technology holds the promise for improving molecular diagnostic tests through miniaturizing techniques. Microfluidics can simplify the procedures, reduce the assay time and sample reagent consumption, avoid the risk of contamination, lessen the unit cost, lower power consumption, and enhance reaction efficiency^1,8–10^. In general, there are two approaches through microfluidics technology by which immunoassay can be improved: (1) enhanced surface immobilization or (2) effective mass transport and diffusion. Between the two, most of the reports focus on increasing the active surface to contact area ratio for increased concentration and kinetics by surface treatment^10,11^ or through the use of microbeads^12^. The latter approach is limited by the relation that suggests that the effective transport rate and the capture rate of the target molecules in the laminar flow have weak dependence to the flow rate^13–15^. When convective transport or flow dominates the diffusion, as in the case of a conventional fluidic channel, only the target molecules that are very close to the active surface can bind with the sensor. The number of molecules captured within the boundary layer can be estimated as:

1 $${J}_{D} \sim {{\rm{W}}}_{{\rm{s}}}{{\rm{c}}}_{0}{(6Q{D}^{2}{L}^{2}/{H}^{2}{W}_{c})}^{\frac{1}{3}}$$

where W_s_ is the sensor width, c_0_ is the sample concentration, Q is the flow rate, D is the diffusivity of the molecules, L is the channel length, H is the channel height, and W_c_ is the channel width. From the equation, to obtain at least twofold increase in capture rate of molecules, an eightfold increase in volumetric flow rate is needed. Conversely, an increased flow rate can still provide a significant decrease in reaction time. In addition, another approach to increase the capture rate is by reflowing the reagents through the channel. A better detection can be done by passing the target-carrying liquid to the active surface repetitively to increase the chance of interaction and binding.

Our group has recently reported a recycling flow driven in a ring-structured channel by centrifugation-controlled convective (C3) flow and has been successfully employed for PCR application^16^. This technique provides a reflow system which could significantly reduce the amount of reagents required to drive the flow. Briefly, by simply altering the relative gravity acceleration, G, the thermal convective flow (Rayleigh–Benard convection) can be controlled following the Boussinesq equation:

2 $$\frac{\partial u}{\partial t}+(u\cdot \nabla )u=-\frac{1}{{\rho }_{r}}\nabla p+v{\nabla }^{2}u-G\alpha (T-{T}_{r})$$

3 $$\frac{\partial T}{\partial t}+(u\cdot \nabla )T=k{\nabla }^{2}T$$

4 $$\nabla \cdot u=0$$

where u is the velocity field, ρr is the standard density, p is the reference pressure, ν is the kinematic viscosity, κ is the body expansion coefficient, α is the thermal diffusivity, and Tr is the reference temperature.

For this report, the same technique has been utilized for on-chip ELISA. The use of centrifugation based microfluidics, aside from ease in multiplex fluid manipulation, has a great commercial potential because of simple instrumentation, ease for economic mass production, and easy adaptation to existing optical detection methods^17–20^. Bubble formation and dead volume are also avoided in centrifugation based microfluidics. Bubbles can affect the device performance for it can disrupt the continuity of the liquids and even the applied electric field in electrophoresis-based microdevices^21^. There are already several reports on centrifugation-controlled immunoassay system that have several integrated multiplexing operation and detection mechanisms and has higher number of parallel tests^19,22^. But to the authors’ knowledge, this is the first time that temperature has been utilized as an additional variable in controlling the flow. A similar motivation to use reflow in immunoassay has also been reported but it requires for the rotor to do several steps of acceleration and deceleration^23^ and pneumatic operation^24^ that are usually difficult to control and stabilize without a programmed control unit. In this study, for a designated temperature difference, a fixed rotation speed can be simply set based on the applied voltage to the rotor. Moreover, the integration of a heat source to control the temperature is also beneficial for certain biochemical reactions such as PCR and enzymatic reaction. The inclusion of the heater or cooler stage can be exploited for long-term storage of bioreagents since some are not suitable and not durable under room temperature and some require special environment for storage^8^. However, this is not explored in the current report.

This report intends to disclose the utility of the C3 flow, the design and the operation of the PDMS microfluidic device that contains the detection microbeads, and the details of the developed rotating heater stage for on-chip ELISA. The device has been tested for IgA detection and could be revised for other immunoassays by simply changing the capture antibodies. With these, an alternative system for ELISA is realized.

## Materials and Methods

### Reagents and materials

The components of the human IgA (Immunoglobulin A) ELISA Quantitation kit set from Bethyl Laboratories Inc. (Montgomery, Texas, USA) were utilized in this study. It contains the affinity purified goat anti-human IgA coating antibody (1 mL at 1 mg/mL), the human reference serum (1.8 mg/mL IgA) and HRP conjugate goat anti-human IgA detection antibody (1 mL at 1 mg/mL). 3, 3′,5,5′- Tetramethylbenzidine (TMB) substrate and stop solution (0.18 M H_2_S0_4_) were obtained from R & D Systems (Minneapolis, Minnesota, USA). Wash solution (0.05% Tween 20) and blocking solution (1% Bovine Serum Albumin, BSA) were prepared using Phosphate-Buffered Saline (PBS, pH 7.4) as diluent. Polydimethylsiloxane (PDMS, Sylgard 184) was purchased from Dow Corning (Midland, MI, USA) while the polystyrene microbeads (250 µm diameter, 25 mg/mL in water) with COOH surface coating was purchased from micromer (Erlinsbach, Switzerland).

### Design and fabrication of the microfluidic chip

**Figure 1:**
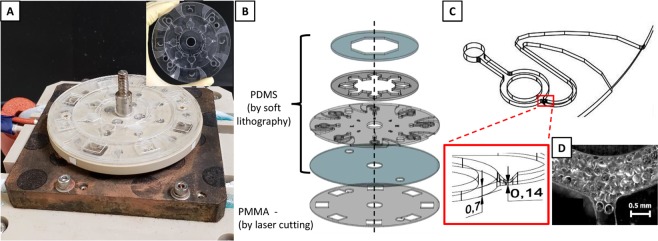
Fabricated microfluidic disk chip. (**A**) The assembled fluidic placed on the heater stage. Inset: microfluidic disk chip. (**B**) The disk consists of four layers of PDMS with PMMA bottom layer as support. (**C**) The ring channel served as the reaction chamber. Expanded image shows the height depression that prevents the (**D**) microbeads carrying the capture antibodies from escaping the main channel.

Figure 1 presents the microfluidic device utilized in the study. It consists of 4 PDMS layers with 1 mm thick Poly(methyl methacrylate) (PMMA) bottom layer as support (Fig. 1B). The PDMS layers were fabricated using the conventional soft lithography technique with polycarbonate (PC) molds as pattern source. The PC molds, shaped by machine cutting process, was used to minimize the dimension variability in the fabricated PDMS as what was observed when using SU-8 molds (data not presented). Figure S1 contains the complete details of the measurements of the molds and the resulting PDMS layers. The first PDMS layer serves as the main inlet and cover of the second layer to prevent the liquid from spilling out of the chambers. The second layer secures and isolates the liquid inside the pentagonal prisms and prevents contamination. The tip of the base pentagon has a 2.4 mm square through-hole that serves as connecting channel to the third layer. The third layer or main layer contains the ring-structured channel with 1 mm width and an average circumference of 6 mm. The fluidic channel in this layer has a height of 0.7 mm and a height depression of 0.14 mm at the main-to-outlet channel junction (Fig. 1C). This functions as a physical barrier to keep the antibody carrying microbeads from escaping the main channel. A U-shaped channel was also utilized at the outlet to control the fluid discharge and the increasing width of the flow path at the end was done to minimize the capillary flow effect. In addition, the PMMA bottom layer has square holes aligned with the circular main channel to avoid obstructing the optical path and subsequently the measured absorption signal.

### Antibody immobilization on microbeads

Desired target antibodies were immobilized on the microbeads using physical adsorption. A volume of 1 mL microbead suspension from the stock was added to a 2 mL Eppendorf tube and the supernatant was removed after centrifugation. A volume of 250 µL capture antibody solution (12 µg/mL in PBS) was added afterwards and reacted for 3 hours in a rotary mixer. The microbeads were then washed for three times (using PBST) and then diluted at desired concentration (in PBS).

### Construction of rotating stage with heaters

**Figure 2:**
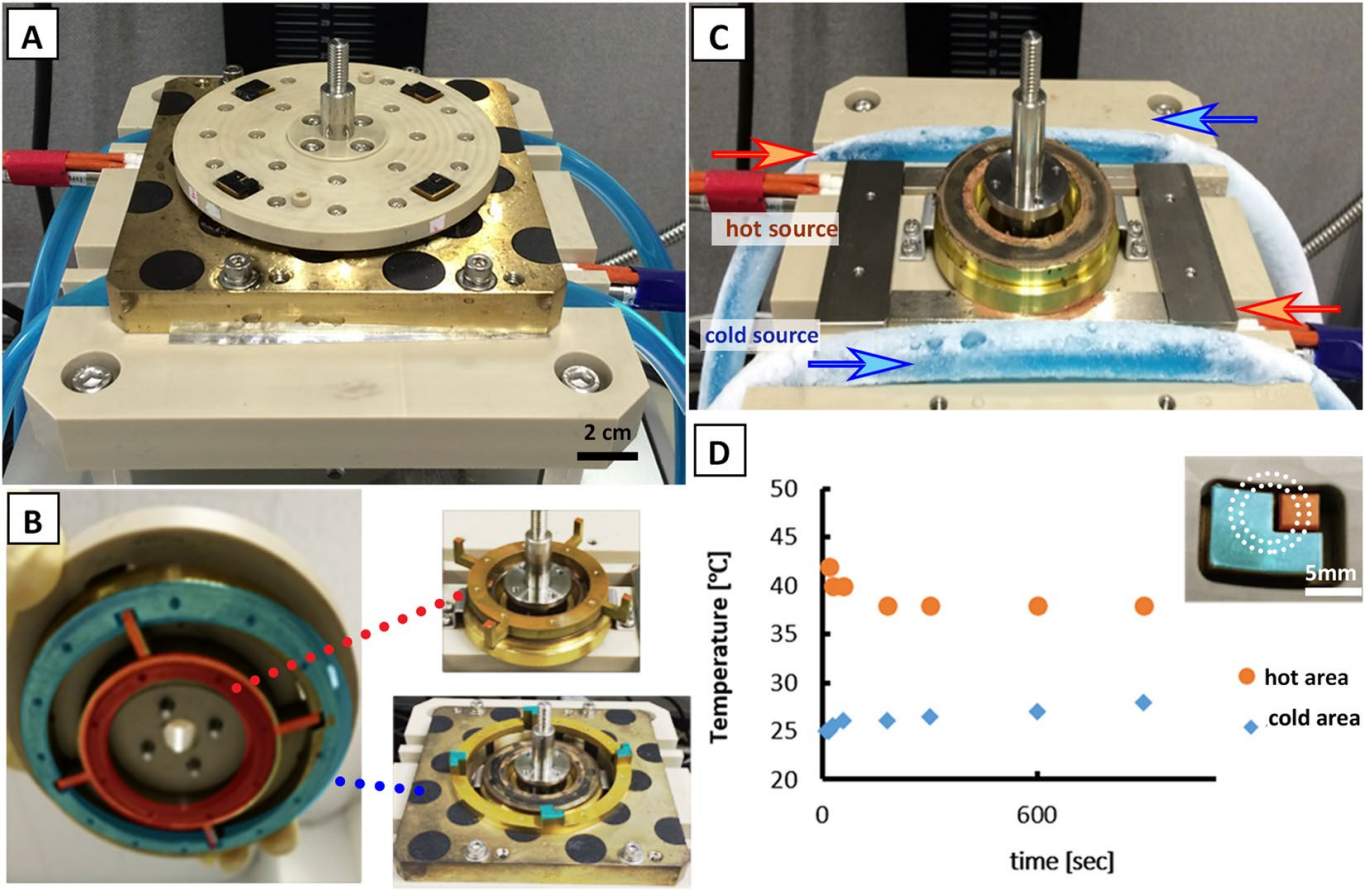
Developed rotating heater stage. (**A**) Actual set-up of the rotating stage with four separate pairs of heaters. (**B**) The high temp (orange) and the cold temp (blue) heaters were in contact with the conducting plates connected to separate heat sources. (**C**) Two pairs of heat sources were placed along the cavities of the fixed stage. (**D**) A stable temperature for the hot heater and the cold heater was observed for 15 min; 38 °C and 24 °C, respectively. Inset shows how the channel was aligned with the heaters.

Figure 2 illustrates the assembled automated rotating heater stage device. It consists of the stage, heat source, and motor (TG 78 A-KA, Tsukasa Electronics Co., Ltd., Tokyo, Japan). The rotating stage (Polyether ether ketone, PEEK) has an integrated heater with conduction lines (brass) arranged as shown in Fig. 2B, which allows heat transfer from two sources separately. For visualization purposes, the hot area was colored orange while the cold area was colored blue. The details on the configuration of the conduction circuit are presented in Fig. S2. The fixed stage (PEEK) has a center hole for the rotor shaft passage and four cavities for heat source lines: two lines for each heater (Fig. 2C). The two lines near the center hole served as the source of the high temperature and were controlled by a digital controller (SR 83, Shimaden, Tokyo, Japan). A heater block was included to ensure the uniform distribution of heat. For the low-temperature source line, a coolant (Nybrine Z1, Nisso Shoji Co., Ltd., Tokyo, Japan) was fed to a 10 mm diameter and 1 mm thick silicone rubber tube at 5 L/min and the desired temperature was set using a chiller (NCB-1200, Eyela, Tokyo, Japan). A conducting plate with a hollow bottom was added that serve as the contact point to the low-temperature source and to avoid physical contact with the high-temperature source. The ring bases of the conduction lines integrated to the rotating stage remained in thermal contact and isolated to each respective heat sources even during rotation. To reduce the friction when moving but still promote the conduction of heat, a thin layer of silicon grease (TK-P3C, Sanwa Supply, Okayama, Japan) was coated to the ring bases. For controlling the rotation speed, the voltage supplied to the motor was regulated by a DC stabilized power supply (PAS 20-18, Kikusui Electronics Corp., Kanagawa, Japan).

### On-chip ELISA test

It was observed that the wettability of the PDMS surface varies within few hours after exposure to O_2_ plasma (Fig. S3). To minimize this effect, an assembled microfluidic chip that was exposed to O_2_ plasma after two days was utilized in the experiment. Fluid flow was controlled by the rotation speed of the developed heater stage. Prior to microbead filling, the channels were blocked with 1% BSA. After washing, 114 µL of antibody-coated microbeads solution at 2.9 × 10^3^/mL concentration was delivered to the microchannel and about 40% of the circular flow path was filled (Fig. 1D). A volume of 24 µL antigen protein solution at various concentration was centrifugally fed to the channels and interacted with the microbeads. The channels were washed with 25 µL PBST solution six times before adding 24 µL of HRP-labeled detection protein solution. After the desired time of reaction, the channels were washed again with PBST six times and the 24 µL substrate solution was added after. Lastly, 4 µL of stop solution was added. The tests were conducted in a controlled environment as shown in Fig. S4 to keep the humidity constant.

After the reaction, the microfluidic chip was removed and set on a microscope stage. The absorbance was then measured at the area of the ring-structured channel without microbeads. The light emitted from the Tungsten halogen light source LS-1 (Ocean Optics, Florida, USA) was adjusted by an ND filter (Edmund Optics, New Jersey, USA) and was irradiated to the target spot. The light transmitted was directed to a spectrometer (USB 4000, Ocean Optics, Florida, USA) and the absorbance spectra graph was generated by a computer (Fig. S5) (integration time of 8 ms and average of 50 readings). Using the Beer-Lambert Law, the relation of the resulting absorbance spectra to the concentration of the analyte was then plotted and the calibration curve was obtained. The absorbance value OD served as the basis to interpret the succeeding concentration measurements.

## Results and Discussion

### Liquid transport and flow control establishment

**Figure 3:**
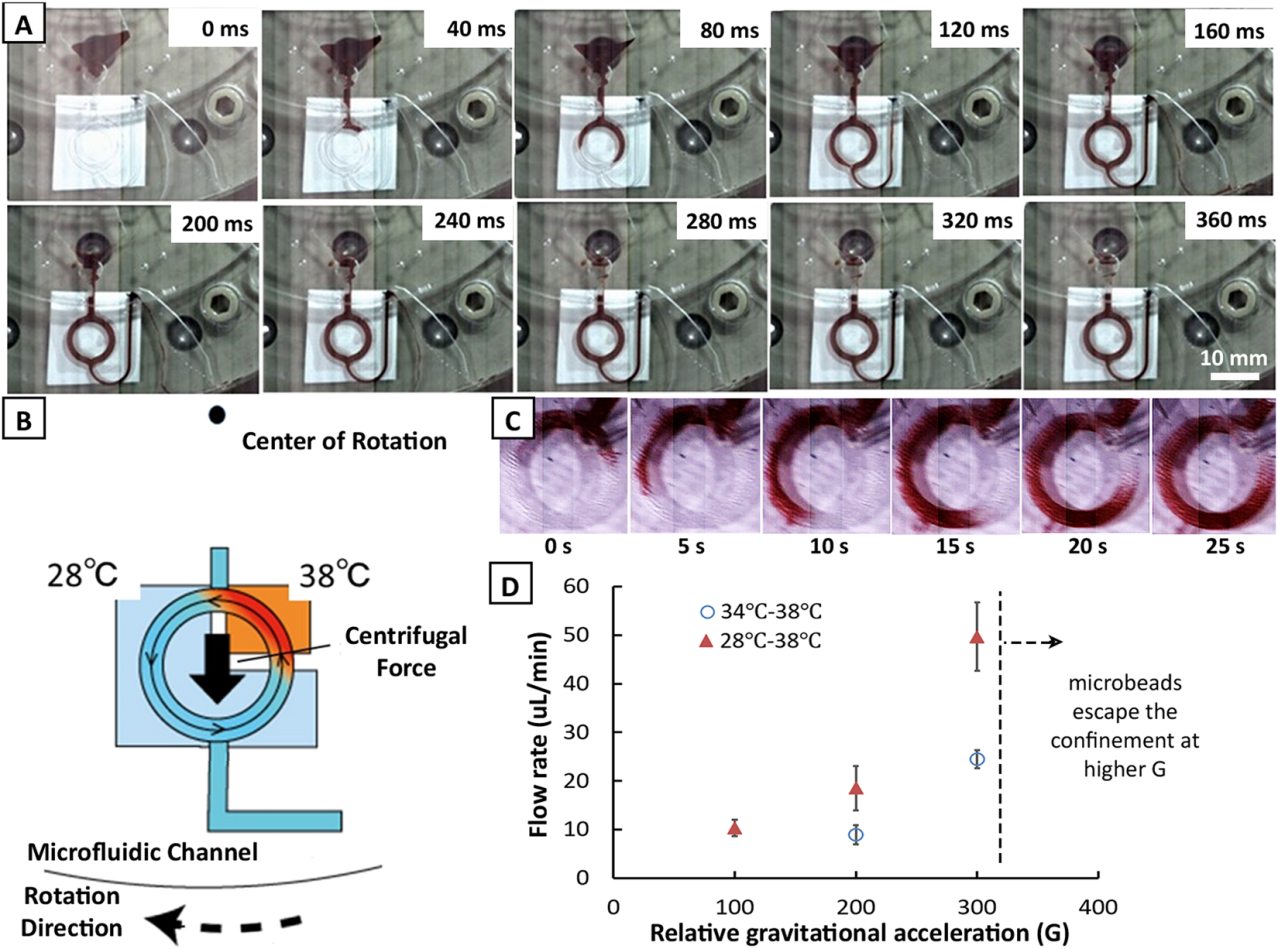
Flow control in the ring-structured channel. (**A**) By centrifugation, the injected fluid was metered inside the ring-structured channel within 360 ms. (**B**) Illustration of the centrifugation-controlled convective (C3) flow inside the channel. (**C**) Observed convective flow under 300 G with a fixed thermal difference of 10 °C. (**D**) Flow rate inside the microchannel at various relative gravitational acceleration G at maximum and at minimum possible thermal difference in the set-up. Error bar represents standard deviation with n = 3.

Figure 3 illustrates the fluid flow control available in the designed microfluidic chip. Fluid exchange and metering can be quickly done by just spinning the microfluidic disk after fluid injection. It can be seen from Fig. 3A that the U-shaped channel meters the amount of fluid in the ring-structured channel along the outlet. This is only available in centrifugation-based microfluidic chips. After at least 360 ms, the channel was filled with the liquid when spun at 1500 rpm. The widened part of the outlet limits the effect of the capillary flow and avoids the siphoning effect when the rotation is suddenly ramped down. In addition, the metering feature of the device doesn’t depend on the rotation speed and the user doesn’t need to be mindful of the volume added. To promote the thermal convective flow in the chip, the channels were aligned with the heaters as shown in Fig. 3B. An example of the observed centrifugation controlled convective (C3) flow is illustrated in Fig. 3C. As what has been reported before, this flow is only available when thermal difference is present and that the direction of the flow is independent of the direction of the rotation of the stage. Currently, the cold source can only reach as low as 28 °C and heat up to 34 °C. A temperature of 38 °C was set at the hot source which is similar to the human body temperature and is expected suitable for antibody-antigen reaction. More importantly, though ELISA is conventionally performed at room temperature, the current working temperature range (28 °C–38 °C) remain suitable for assay condition. A stable set temperature can be observed for 15 min as seen in Fig. 2D and a uniform heat distribution was verified using a thermal seal as shown in Fig. S6. This minimizes the measurement error associated with the machine. Thus, a stable and relatively high flow rate is expected. Fig. 3D shows the observed flow rate at extreme possible settings of temperature difference for the heat sources. As expected, the higher temperature difference will result to a faster flow rate when a constant relative gravitation acceleration (G) was applied. Note that 100 G resulted to the same flow rate for both temperature differences. A faster rotation is indeed ideal for more rapid flow rate; however, the rotor can only be set to 4120 rpm at max. Also, it was observed that above 3880 rpm (above 300 G), the microbeads escaped the confinement. Thus, for the succeeding experiments, this rotation speed limit was set. The corresponding flow rate was observed to be 50 µL/min which is considered sufficient to enhance the effective transport rate.

### Centrifugation-controlled convection for on-chip ELISA

**Figure 4:**
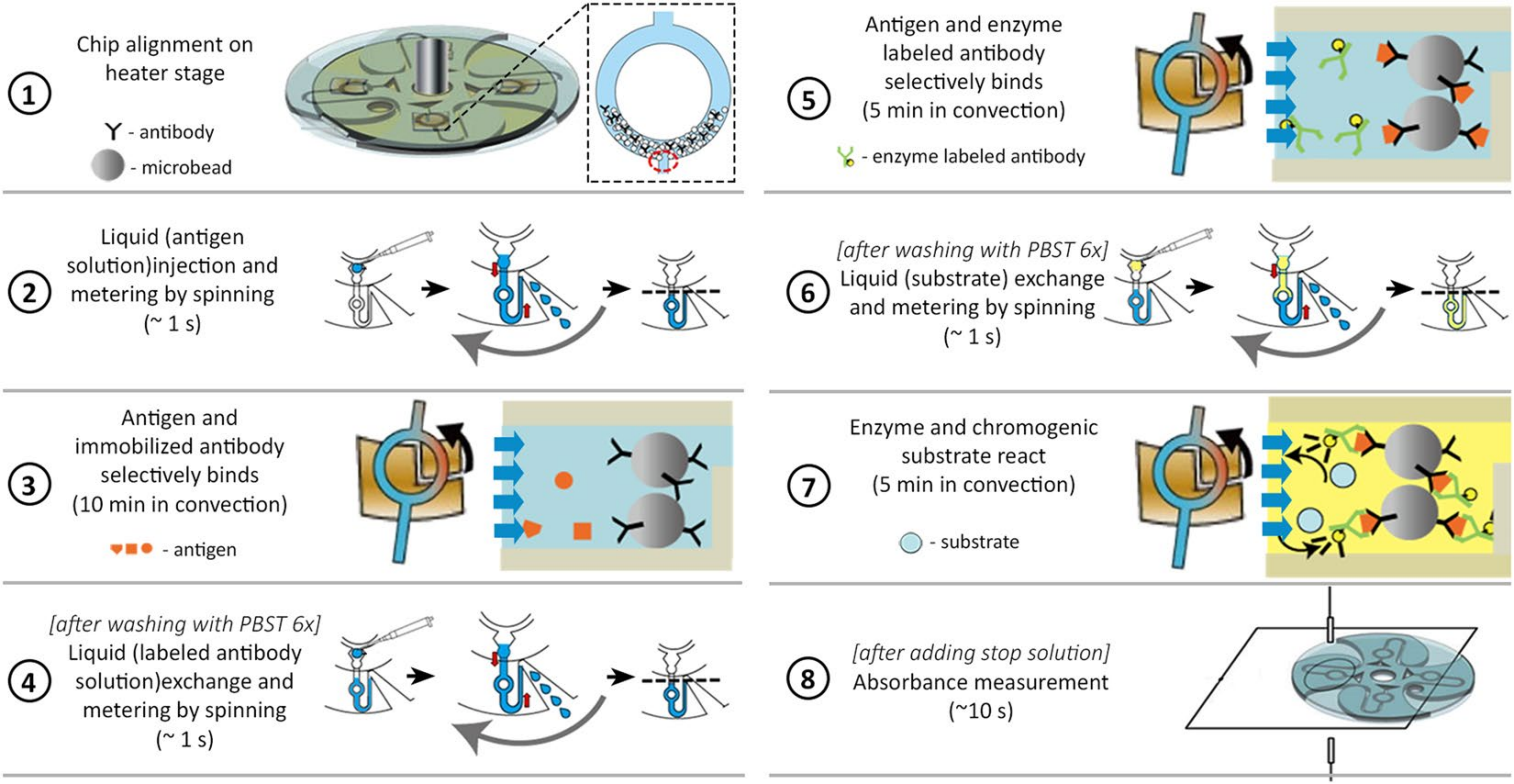
Resulting simplified protocol for on-chip Enzyme-Linked Immunosorbent Assay (ELISA) using C3 flow. The U-shaped channel allows the easy exchange of the solution within the ring-structured channel by automatically metering the injected solution and flowing the excess (dashed line). The induced reflow with the ring-structured promotes a more efficient and rapid reaction.

After determining the rotation speed and the temperature setting available, the operation time of the device was then optimized. The reaction time was determined based on the observed onset of signal saturation (data not included). Figure 4 illustrates the resulting simplified processes involved in operating the system. First, the ring-structured channels blocked with 1% BSA were filled up to 40% with 250 µm microbeads carrying the detection antibodies. The space without microbeads along the ring-structured channel served as observation spot during signal measurement. Originally, it was desired to fill the ring-structured channel with microbeads up to 50% for a higher number of surface sites. However, it was observed that the microbeads start to escape the confinement above 40%. After loading the microbeads, the chip was then aligned to the heaters and the antigen-containing solution was injected. For 1 s, the chip was rotated at 1500 rpm and then ramped up to 3880 rpm. For 10 min, the chip continues to spin while the convective flow was generated, which promotes the antigen-antibody reaction. The amount of the solution present in the channels remains constant while convective flow occurs. The rotation speed was then ramped down to a stop and the enzyme-conjugated antibody solution was added. For another 1 s, the chip was rotated at 1500 and an exchange and metering of liquid were automatically performed. The convective flow was again utilized for 5 min before ramping the rotation speed to a stop. The last liquid exchange was done to introduce the substrate. Similarly, the convective flow assisted the reaction inside the channel for another 5 min. After adding the stop solution, the absorbance spectra were measured. Note that prior to every fluid exchange, washing with PBST (6 times) was done.

It’s important to point out that the convective flow is not strong enough to drive the movement of the microbeads along the circular flow path. The size of the microbeads was crucial in keeping a closed-pack arrangement of the spheres inside the channel and maintaining a relatively higher density (1.2 g/mL) than the surrounding liquid. The sedimentation effect of the centrifugal force keeps the microbeads stable and immobile. This is vital in ensuring that the fluid interacts with the surfaces of the microbeads and promotes the interaction wanted. Moreover, aside from providing a larger surface area compared to the two-dimensional platforms, the microbeads served as a natural mixer that could have promoted to a better binding rate. In a 0.7 mm × 1 mm cross section of the channel filled with microbeads, an estimated 1.3 increase in flow velocity is expected (11 closed-packed spheres) across the gaps between the beads.

Currently, four simultaneous measurements can be performed based on the available integrated heaters. Since the disk contains 8 channels, the remaining four channels can be used as a comparison for non-convective flow or another run can be performed by repositioning the channels along the heaters; rotate the disk by 45°.

### Demonstration and performance of on-chip ELISA

The system was used in quantifying IgA that is responsible for defense against infections respiratory and intestinal mucosa. It can also be a non-invasive marker for stress. The normal concentration of IgA is 3 mg/mL in blood and could also be found in exocrine fluids (saliva, tears, bronchial fluid, breast milk, etc.). Thus, in actual measurements, dilution of the real sample will be needed depending on the working detection range of the device.

**Figure 5:**
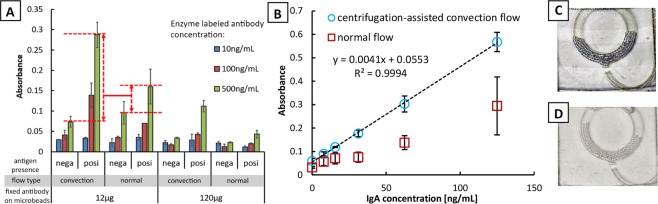
Detection test using IgA. (**A**) Difference of the measurements based on convective flow and normal diffusion. Comparison of absorbance measurement using different amounts of fixed detection antibody on the microbeads and of enzyme-labeled antibody solution is also presented. The negative control of antigen sample was included to consider the effect of non-specific adsorption and determine the threshold. The highlighted region indicates the 1.66-fold signal enhancement. (**B**) Calibration curve of on-chip ELISA using C3 flow for IgA. Error bars represent standard deviation with n = 5. Visible difference of (**C**) 125 ng/mL and (**D**) blank, 0 ng/mL after adding the stop solution.

The ideal amount of antibody-coated to the detection beads was determined by determining the maximum absorbance obtained after reacting with the enzyme-labeled antibody at a constant IgA concentration or target antigen (62.5 ng/mL). Figure 5A summarizes the results. Negative control for the antigen sample was included to consider the effect of non-specific adsorption of the enzyme-labeled antibody to the microbeads. Two amounts of immobilized antibody on microbeads were prepared: 12 µg and 120 µg. The result shows that 12 µg gives a stronger signal than 120 µg. The antibodies at a higher concentration must have been tightly bound with one another which limits the interaction with the target antigen. For the concentration of the enzyme-labeled antibody, three solutions were tested; 10 ng/mL, 100 ng/mL, and 500 ng/mL. A concentration of 500 ng/mL produced the strongest difference in the signal of the positive and negative control. From this result, it was decided to adopt the 12 µg as the amount of the antibody fixed to the microbeads and 500 ng/mL as the enzyme-labeled antibody concentration.

The signal enhancement effect of the centrifugation-controlled convective (C3) flow compared to the normal diffusion flow is also presented in Fig. 5A. The measurement for the normal diffusion is based on the same system but without application of heat. It can be seen that a significant increase in the signal was obtained; 1.66-fold improvement in signal. Besides, a smaller variability was observed for experiments using the convective flow compared to the typical diffusion flow. This increase in detection grades and lesser variability lead to a better and more accurate measurements that are warranted in any biosensing platforms.

Figure 5B presents the resulting calibration curve for IgA. The ELISA C3 flow system has a detection limit of 6.16 ng/mL with an analytical sensitivity of 0.0041 a.u.mL/ng and an R^2^ value of 0.9994. A linear range of 6.16 ng/mL–125 ng/mL was determined with residual sum of squares (RSS) of 0.001. Above 125 ng/mL, the measurement had a high variability which could be associated to the number of microbeads confined in each channels. Although careful attention was given in keeping the number of microbeads constant while inserting into the channel, the actual number trapped in the channel can’t be kept uniform with the current set up. Table S2 summarizes the recovery test in detecting IgA from human reference serum. The values were close to the spiked ones, together with high detection recoveries of 98.78–104.5% and low RSD of 1.71–3.46% (n = 5). This indicates a good correlation with the detected signal to the set measurements. Also, an increase (~2x) in sensitivity was observed compared to the normal diffusion flow based from the slopes. A higher limit of quantification is expected from the data obtained in the normal diffusion but determining the value is proven difficult due to the large variability.

In comparison to the conventional ELISA plate, a detection limit of 0.179 ng/mL was observed which was lower than that of the current device and with detection range reaching up to 500 ng/mL. The OD value of the microfluidic chip is expected to be higher than the plate reader. Thus, the sensitivity of the device could be further improved by using a different material or by fabricating a thinner fluidic device. More importantly, the conventional method requires at least 135 min to perform; whereas, the ELISA C3 flow only takes 30 min.

With this result, it has been demonstrated that the developed system has been successfully utilized in improving ELISA by decreasing the reaction time and the operation time but still with relatively good sensitivity.

## Conclusion

A novel approach for decreasing the reaction time and operation time of the centrifugal microfluidic ELISA through reflow of analytes in a ring-structured channel using the centrifugal controlled convective (C3) flow has been demonstrated. The system allows the automatic metering of the reagents used in the tests with the help of the microchannels design and structure. The developed rotating stage with integrated heaters has been proven valuable in providing a constant heat source while the microfluidic disk is in motion. The heat sources can be utilized later as cooling pads for storing delicate bioreagents to conduct the test for on-site measurement. More importantly, the fluid flow is simply manipulated based on rotating speed. This promotes the ease of operation without the need for specialists to control. In the current work, detection of IgA was successfully demonstrated with a detection limit of 6.16 ng/mL using 24 µL of the target sample. The target-antibody reaction was 10 min, the enzyme-labeled antibody reaction was 5 min, and the substrate reaction time was 5 min in the convective flow system. It can be revised for other immunoassays by simply changing the capture antibodies. In addition, the current sensitivity and detection limit could be further enhanced by adopting other techniques in minimizing the nonspecific binding on the surfaces or utilizing other materials aside from PDMS.

## Supplementary information

Supplementary information.
