# Supplementary material for: Utility of Centrifugation-Controlled Convective (C3) Flow for Rapid On-chip ELISA
Source: Sci Rep. 2019 Dec 27;9:20150. doi: 10.1038/s41598-019-56772-6 (PMC6934823; doi:10.1038/s41598-019-56772-6)
Supplement: Supplementary file 1 — Supplementary information. [file 41598_2019_56772_MOESM1_ESM.docx]

**Utility of Centrifugation-Controlled Convective (C3) Flow for Rapid On-chip ELISA**

Wilfred Espulgar^1^, Tatsuro Tadokoro^1^, Eiichi Tamiya^1^ and Masato Saito^1,2^*

^1^Department of Applied Physics, Osaka University, 2-1 Yamadaoka, Suita, 565-0871, Japan

^2^AIST, PhotoBIO-OIL, Japan

*saitomasato@ap.eng.osaka-u.ac.jp

**Supplementary Materials**


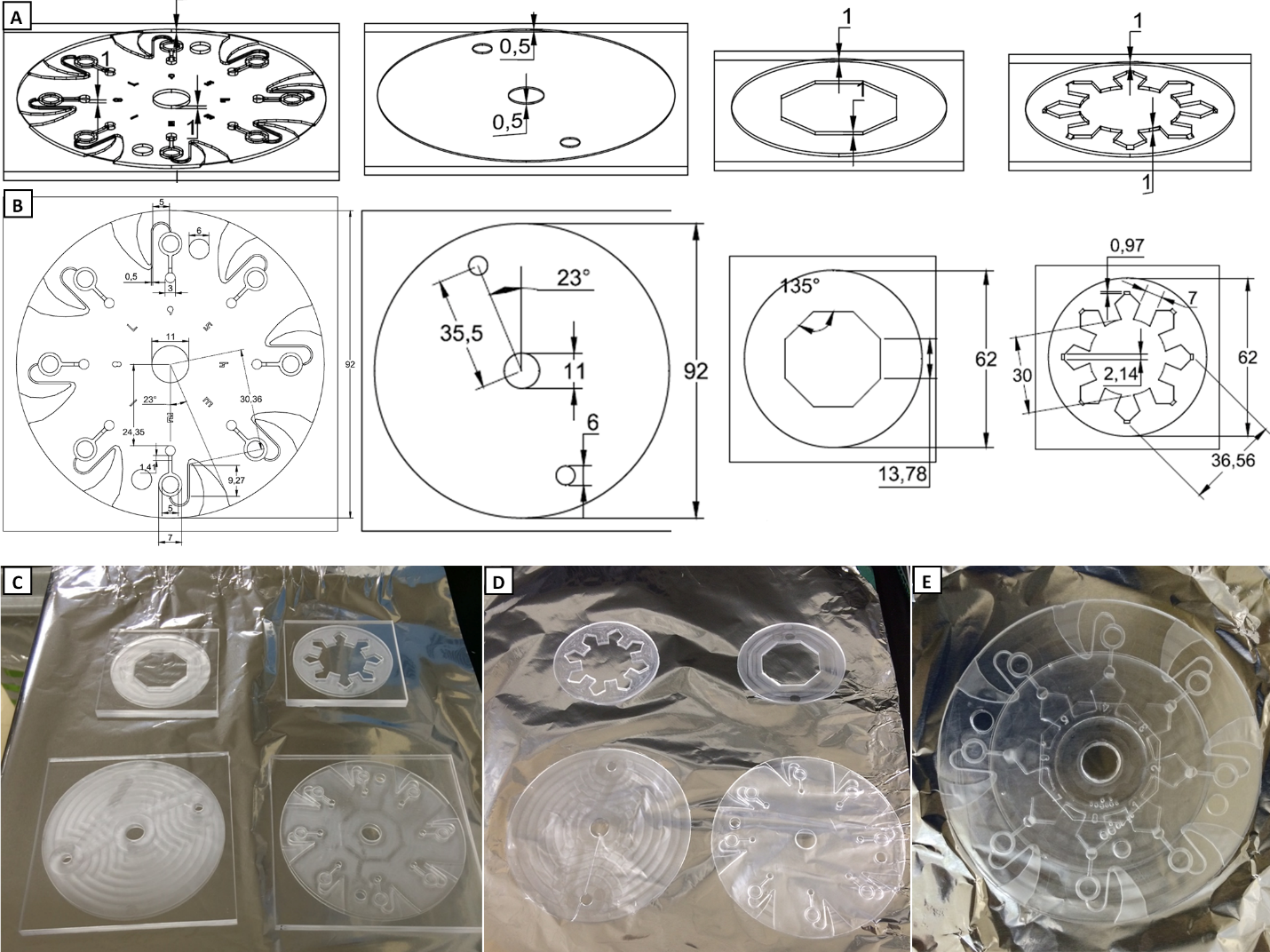


**Figure S1.** Microfluidic disk design and fabricated PDMS layers. (A) 3D view and (B) top view of the wire frame illustration of the design. Measurements are in mm. (C) Polycarbonate molds. (D) PDMS layers. (E) Assembled microfluidic disk.


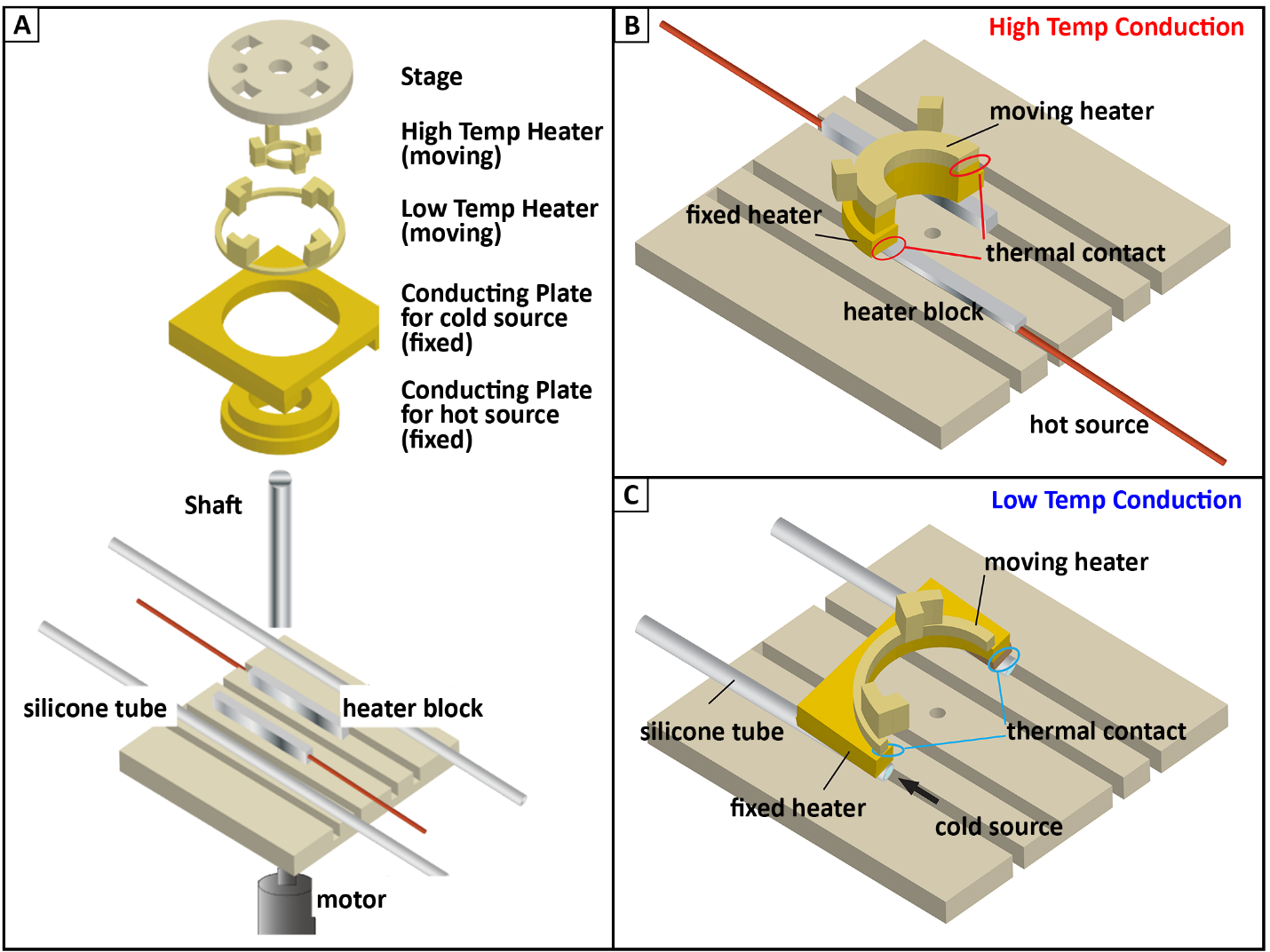


**Figure S2.** Schematics of conduction line of the developed heater stage. (A) Composition and arrangement of the rotating heater stage and the fixed heater stage. (B) Conduction line for high-temp source. (C) Conduction line for low-temp source.


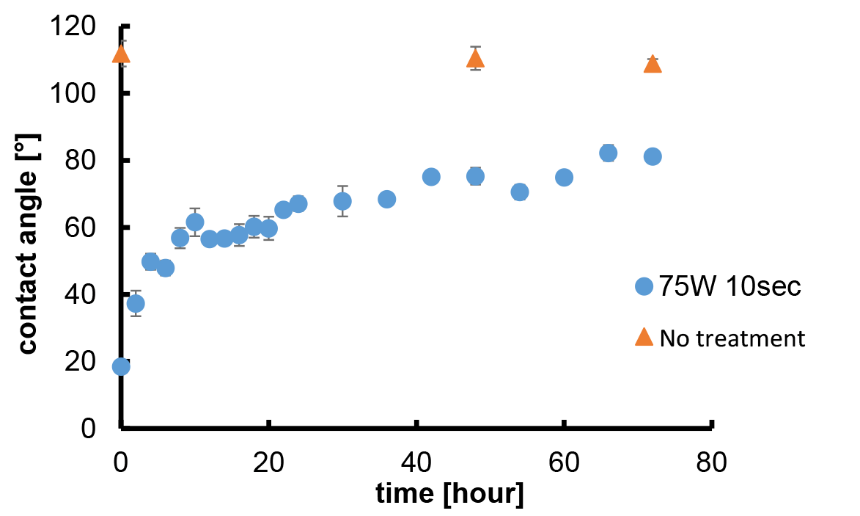


**Figure S3.** Contact angle of water on PDMS surface after O2 plasma treatment. No treatment served as reference. A relatively stable and lesser change in the contact angle was observed for a PDMS surface 2 days after O2 plasma exposure. (n=3)


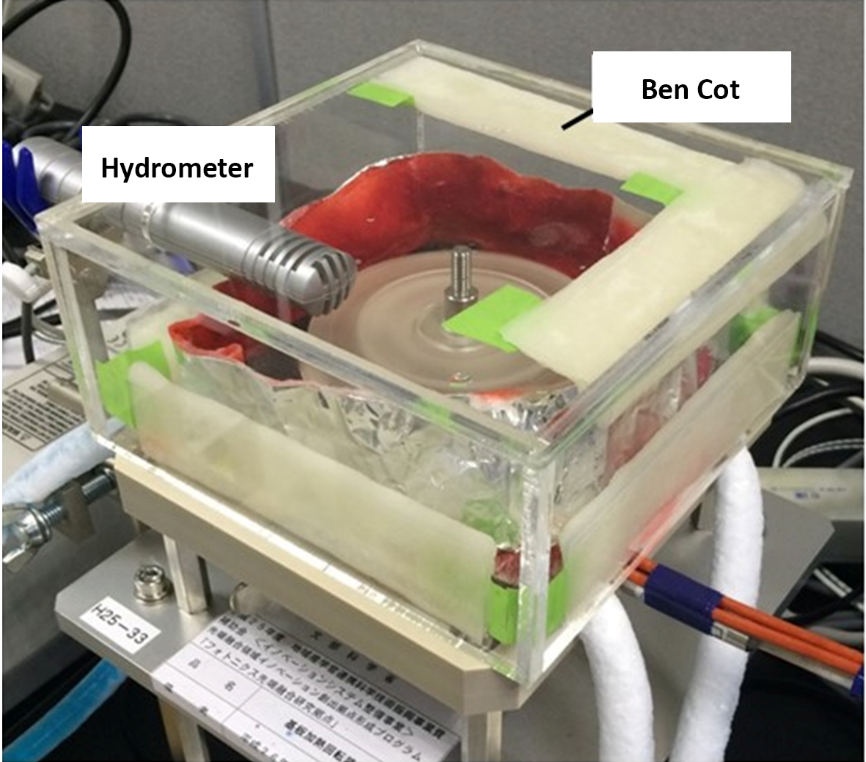


**Figure S4.** Controlled set-up for ELISA tests. The humidity was kept constant in the PMMA chamber.


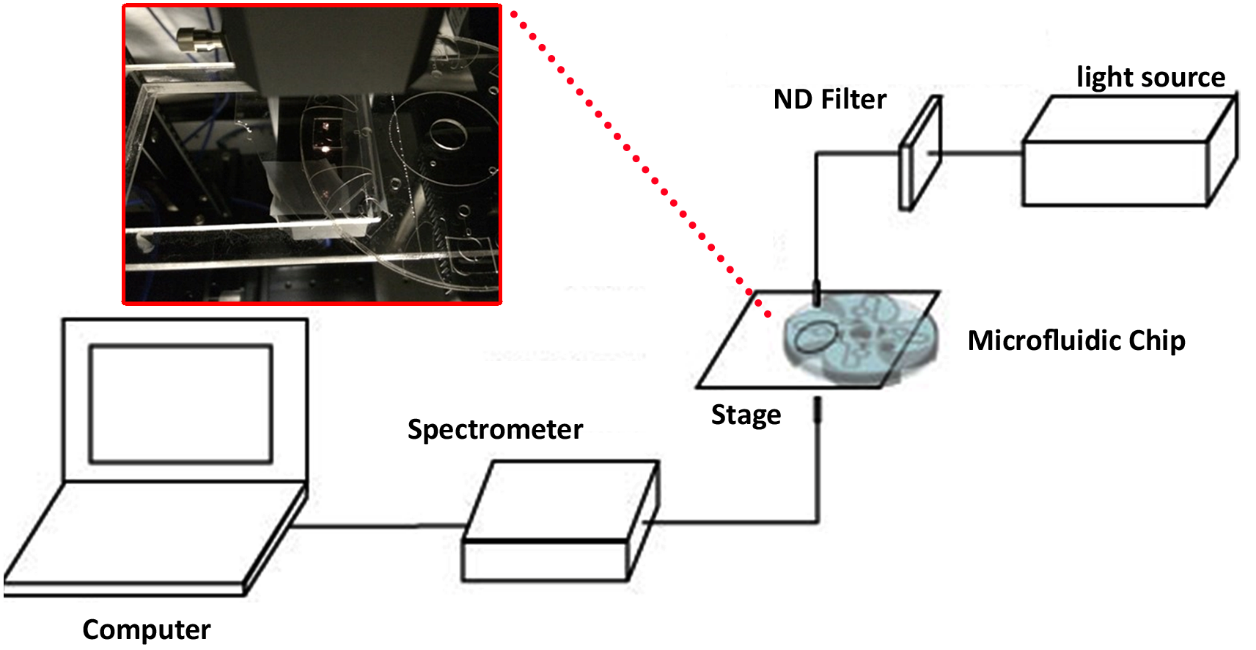


**Figure S5.** Schematics of absorbance measurement. Inset shows the actual arrangement.


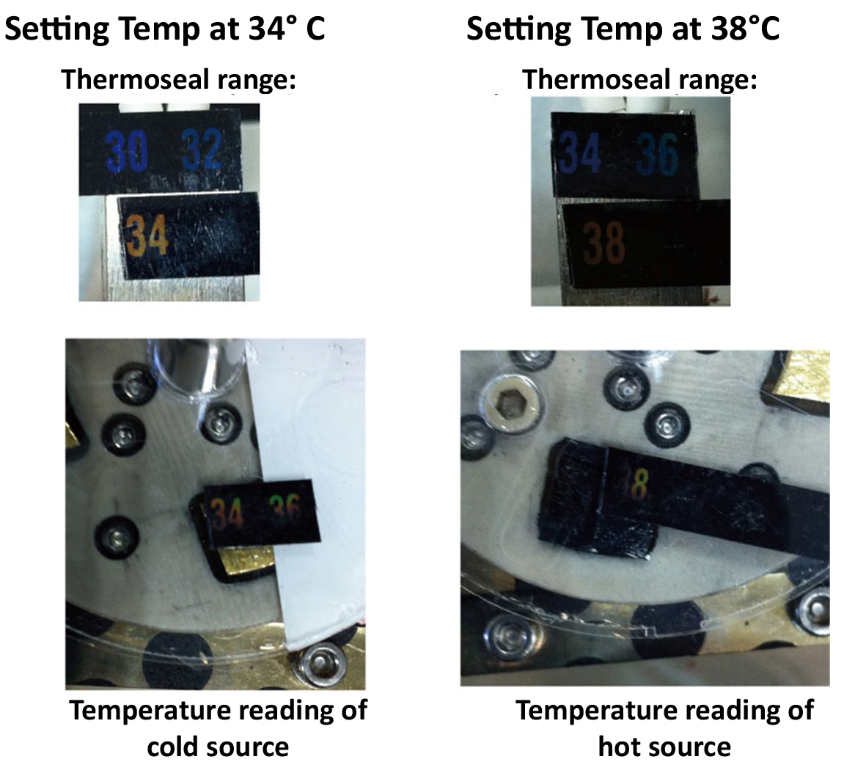


**Figure S6.** Set thermoseal on the low temp and the high temp heaters.

**Table S1**

Detection of IgA in from human serum samples provided in the kit by means of this sensor.

| ***^a^* Sample** | **Spiked /nM** | ***^b^* Detected /nM** | ***^c^* RSD /%** | **Recovery /%** |
| --- | --- | --- | --- | --- |
| #1 | 0 | Not found | – | – |
| #2 | 10 | 10.45 ± 0.18 | 1.72 | 104.5 |
| #3 | 50 | 53.20 ± 0.91 | 1.71 | 106.4 |
| #4 | 100 | 98.78 ± 3.42 | 3.46 | 98.78 |

*^a^* Samples were prepared from real human serum samples with PBS (1 mM, pH 7.4).

*^b^* All detected results were expressed as the average of 5 repeated determinations ± standard deviation (SD).

*^c^* Relative standard deviation (RSD) was defined as (SD/mean) × 100%.
